# Numerical Simulation and Experimental Study on Polishing Fluid Dynamics and Material Removal in Metal Ultrasonic Vibration Polishing

**DOI:** 10.3390/mi17020208

**Published:** 2026-02-03

**Authors:** Xianling Li, Jingchang Chen, Dalong Zhang, Bicheng Guo, Xiuyu Chen, Zhilong Xu

**Affiliations:** College of Marine Equipment and Mechanical Engineering, Jimei University, Xiamen 361000, China; 15960315013@163.com (J.C.); zhangdalong0303@163.com (D.Z.); guobicheng@jmu.edu.cn (B.G.); jdcxy@126.com (X.C.); zhilong.xu@163.com (Z.X.)

**Keywords:** computational fluid dynamics (CFD), material removal mechanism, ultrasonic vibration polishing, cavitation, polishing fluid dynamics

## Abstract

To address the bottleneck issues of traditional ultrasonic polishing—such as unclear material removal mechanisms for ductile metals and difficulties in controlling machining outcomes—this paper employs a combined approach of computational fluid dynamics (CFD) simulation and non-contact fixed-point polishing experiments to systematically reveal the intrinsic relationship between the dynamic characteristics of the polishing flow field and the evolution of the material surface. Numerical simulations demonstrate that the cavitation effect significantly regulates the flow field structure: it not only confines the minimum pressure near the saturated vapor pressure but also markedly reduces the pressure peak while concurrently causing an overall decrease in flow velocity, forming a strongly coupled multi-parameter system of pressure, cavitation, and flow velocity. Experimental results indicate a clear spatial differentiation in the material removal mechanism: the central region is dominated by cavitation erosion, resulting in numerous pits and a 33.6% increase in residual compressive stress; the edge region is primarily governed by fluid-mechanical scraping, effectively improving surface finish and increasing residual stress by 22.3%; the transition zone, influenced by synergistic mechanisms, shows the smallest stress increase (19.7%). The enhancement of residual compressive stress can significantly improve the fatigue resistance of materials and prolong their fatigue life. This study comprehensively elucidates the multi-mechanism synergistic material removal process involving “cavitation impact, mechanical scraping, and fatigue spallation” in ultrasonic polishing, providing a key theoretical basis and process optimization direction for sub-micrometer ultra-precision machining.

## 1. Introduction

Polishing is a key finishing process for achieving high-quality surfaces and is widely used for metallic materials; however, conventional methods are increasingly limited by material variations and the growing demand for higher efficiency and better surface quality [1]. With the introduction of ultrasonic machining, ultrasonic-assisted polishing leverages cavitation and mechanical vibration to markedly improve the material removal rate and surface quality [2,3,4,5] and can be readily integrated into CNC systems with high efficiency and minimal surface damage.

Ultrasonic vibration polishing dates back to the 1920s and has shown clear advantages in machining hard and brittle materials [6,7]. Studies indicate that ultrasonic assistance can improve surface morphology and increase the material removal rate, enabling both high efficiency and high surface quality in processing cemented carbide molds and monocrystalline silicon [8,9,10]. Material removal mainly involves cavitation, abrasive impacts, and microscale grinding; integrating ultrasonic vibration with chemical–mechanical polishing and optimizing process parameters can further achieve high-efficiency, high-quality finishing [11,12,13,14].

Current studies on ultrasonic abrasive polishing mainly focus on process parameters, machining quality, and material removal rate, whereas the dynamic behavior of the polishing fluid in ultrasonic flow fields has received less attention. Material removal primarily relies on cavitation and abrasive grinding under ultrasonic excitation [15,16,17]. Ultrasonic vibration induces periodic fluctuations in pressure, velocity, and vapor volume fraction, driving complex fluid-abrasive motion that promotes removal; therefore, investigating the polishing fluid dynamics is essential for clarifying the removal mechanism.

Computational fluid dynamics (CFD) is an effective tool for studying the complex hydrodynamic behavior in ultrasonic polishing [18,19]. Skoczypiec S et al. and Sajjadi B et al. used CFD simulations to investigate fluid dynamics under ultrasonic excitation, showing that ultrasonic vibration can alter the flow and cavitation conditions in the processing gap and thereby increase the material removal rate [20,21]. Guo Z et al. combined CFD and experiments to clarify the material removal characteristics in ultrasonic polishing of BK7 optical glass and found that removal due to cavitation bubble collapse is relatively uniform, whereas erosion-induced removal is mainly concentrated in high-velocity regions [22]. Yu T et al. analyzed the polishing fluid flow using CFD and indicated that material removal primarily depends on the shear flow generated near the workpiece surface [23]. In addition, Qi H et al. proposed a suspension polishing method based on numerical simulations, providing guidance for process-parameter optimization [24]. Recent studies have further explored process-parameter optimization and learning-based control strategies for polishing to improve material removal efficiency and surface roughness [25,26].

Research on ultrasonic polishing has mainly focused on brittle materials, while the removal behavior of ductile metallic materials remains relatively underexplored. Therefore, this study combines CFD simulations with fixed-point polishing experiments and investigates the material removal mechanism of ductile metals in ultrasonic polishing by comparing the post-processed surface morphology and residual stress distribution of the workpiece.

## 2. Model Establishment

### 2.1. Ultrasonic Abrasive Polishing Method

**Figure 1:**
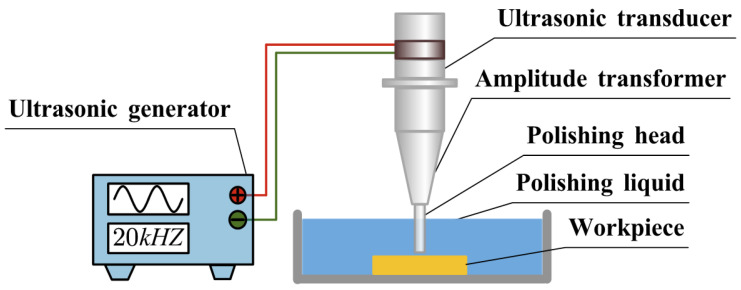
Ultrasonic abrasive polishing system.

The ultrasonic polishing system mainly comprises an ultrasonic power supply, a transducer, an amplitude transformer, and a tool head [27]. During operation, the power supply converts a low-frequency electrical signal into a high-frequency signal. The transducer subsequently converts the electrical signal into mechanical vibrations of the same frequency through the piezoelectric effect. The amplitude transformer amplifies these vibrations, which are then transmitted to the polishing slurry by the tool head, thereby enabling polishing of the target area. A schematic of the overall system is shown in Figure 1.

**Figure 2:**
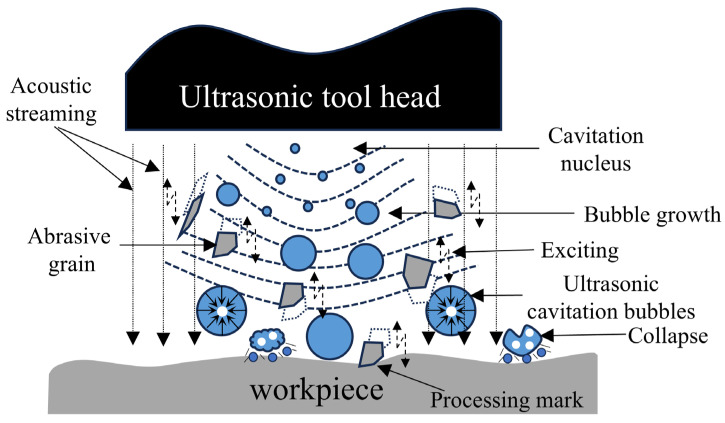
Cavitation phenomenon in ultrasonic abrasive polishing process.

The polishing fluid utilized in ultrasonic abrasive polishing is an abrasive suspension, primarily composed of a base fluid, abrasive particles, and surfactants [28]. Under the influence of ultrasonic vibrations, turbulence and cavitation phenomena are generated within the polishing zone, facilitating uniform and disordered material removal [29]. Cavitation fundamentally involves the growth, contraction, and collapse of air dissolved in the polishing fluid due to localized pressure fluctuations. This process generates high-speed micro-jets that cause cavitation erosion on the material surface. Concurrently, driven by these micro-jets, abrasive particles impart a scratching effect on the workpiece surface [30,31], as shown in Figure 2. Ultimately, the synergistic action of the turbulence and cavitation effects collectively enables material removal within the processing zone.

### 2.2. Multiphysics Coupling Model

This study focuses on ultrasonic cavitation and fluid dynamic behavior during ultrasonic abrasive polishing. Accurately capturing the evolution of cavitation bubbles, interphase interactions, and variations in flow field parameters in numerical simulations requires the integration of multiple mathematical models, including turbulence, multiphase flow, and cavitation models.

#### 2.2.1. Multiphase Flow Model

Two main approaches are commonly used to model multiphase flows: the Euler–Euler method and the Euler–Lagrange method [32]. In ANSYS FLUENT 2021 R1, the available multiphase models include the volume of fluid (VOF), mixture, and Eulerian models, which are all based on the Euler–Euler framework. Considering the cavitation characteristics and flow dynamics in the present process, the mixture model was selected, consistent with the findings of Ni et al. [33].

The mixture multiphase flow model is a simplified multiphase flow solution method based on the continuum hypothesis, suitable for simulating complex multiphase flow simulations where the volume fraction of the dispersed phase is high, e.g., particles, bubbles, and droplets, and slip velocity exists between phases [34].

The governing equations for the mixture multiphase flow model are as follows:

Continuity equation:

(1) $$\frac{\partial }{\partial t}(\rho_{m})+▽\cdot (\rho_{m}V_{m})=0$$

where $\rho_{m}$ = $\sum \alpha_{k}\rho_{k}$ is the mixture density, $\alpha_{k}$ is the volume fraction of each phase, and $V_{m}=\frac{\sum \alpha_{k}\rho_{k}V_{k}}{\rho_{m}}$ is the mass-averaged velocity.

Momentum equation:

(2) $$\frac{\partial }{\partial t}(\rho_{mV_{m}})+▽\cdot (\rho_{m}V_{mV_{m}})=-▽p+▽\cdot \tau_{m}+\rho_{m}g+F_{drag}$$

where p is the shared pressure field; $\tau_{m}$ is the viscous stress tensor of the mixture phase; and $F_{drag}$ is the interphase force source term (e.g., drag force, lift force, and virtual mass force).

Phase volume fraction equation:

(3) $$\frac{\partial }{\partial_{t}}(\alpha_{k}\rho_{k})+▽\cdot (\alpha_{k}\rho_{k}V_{m})=-▽(\alpha_{k}\rho_{k}V_{dr,k})$$

where $V_{dr,k}$ is the drift velocity of the *k*-th phase relative to the mixture.

#### 2.2.2. Cavitation Model

When the mixture multiphase flow model is adopted, a cavitation model can be coupled with the two-phase simulations to account for phase change. In ANSYS FLUENT, the Full Cavitation Model developed by Singhal et al. [35] is used. This model is based on the Rayleigh–Plesset equation describing bubble dynamics and is commonly applied to simulate cavitation inception and development when the local liquid pressure drops below the saturated vapor pressure. It has been widely used in engineering applications such as marine propellers, pumps and valves, and fuel injectors [36].

Rayleigh–Plesset equation:

(4) $$R\frac{d^{2}R}{dt^{2}}+\frac{3}{2}{\left(\frac{dR}{dt}\right)}^{2}=\frac{p_{v}-p_{\infty }}{\rho_{l}}+\frac{p_{g}}{\rho_{l}}{\left(\frac{R_{0}}{R}\right)}^{3\gamma }-\frac{2\sigma }{\rho_{l}R}-\frac{4\rho_{l}}{\rho_{l}R}\frac{dR}{dt}$$

The Rayleigh–Plesset equation defines the relationship between the bubble radius and factors such as pressure and surface tension. Where R(*t*) represents the bubble radius (varying with time); $p_{v}$ represents the saturated vapor pressure of the liquid; $p_{\infty }$ represents the hydrostatic pressure of the liquid far from the bubble; $p_{g}$ represents the pressure of the noncondensable gas inside the bubble (initially $p_{g}(R_{0})=p_{v}+\frac{2\sigma }{R_{0}}$); $\rho_{l},{\;\rho }_{l}$ represents the density and dynamic viscosity of the liquid; σ represents the surface tension coefficient; and γ represents the polytropic index of the gas (γ = 1 for isothermal processes, γ = 1.4 for adiabatic processes).

The governing equations of the full cavitation model proposed by Singhal et al. [37] are as follows:

Continuity equation:

(5) $$\frac{\partial \rho }{\partial t}+\frac{\partial }{\partial x_{i}}(\rho u_{i})=0$$

Momentum equation:

(6) $$\frac{\partial (\rho u_{i})}{\partial t}+\frac{\partial }{\partial x_{i}}(\rho u_{i}u_{j})=-\frac{\partial p}{\partial x_{i}}+\frac{\partial }{\partial x_{i}}\left[\left(\mu +\mu_{t})(\frac{\partial u_{k}}{\partial x_{j}}+\frac{\partial u_{j}}{\partial x_{k}}\right)\right]+\rho g_{i}$$

Vapor mass fraction:

(7) $$\frac{\partial (\rho f)}{\partial t}+\frac{\partial }{\partial x_{j}}(\rho u_{i}f)=\frac{\partial }{\partial x_{j}}\left[(\mu +\mu_{t})\frac{\partial f}{\partial x_{j}}\right]+R_{e}+R_{c}$$

Among these, the density of the mixture is a function of the liquid density, the incompressible gas density, and the vapor density:

(8) $$\frac{1}{\rho }=\frac{f_{v}}{\rho_{v}}+\frac{f_{g}}{\rho_{g}}+\frac{1-f_{g}-f_{v}}{\rho_{t}}$$

The full cavitation model comprehensively accounts for all relevant factors, including phase changes, cavitation bubble dynamics, turbulent pressure fluctuations, and incompressible gas. This model is capable of simulating multiphase flow and mass transport, incorporating the effects of slip velocity between liquid and vapor phases, as well as thermal effects and compressibility of both phases [38].

#### 2.2.3. Turbulence Model

Fluid flow regimes are generally classified as either laminar or turbulent. Reynolds’ experiments demonstrated that when the Reynolds number (*R_e_*) exceeds a critical value, the flow transitions from laminar to turbulent and becomes chaotic [39]. In turbulent flow, local physical quantities such as velocity and pressure exhibit irregular and stochastic fluctuations, distinguishing it from laminar flow [40].

During ultrasonic abrasive polishing, the periodic vibration of the tool head induces cyclic variations in velocity and pressure in the surrounding fluid, which can generate localized turbulent regions near the tool periphery. Therefore, the selection of an appropriate turbulence model is essential for accurately simulating the flow field.

Since no single turbulence model is universally applicable to all problems, model selection requires comprehensive consideration of factors such as fluid compressibility, accuracy requirements, computational resources, and time constraints. Considering the coupled effects of dynamic mesh motion and cavitation phase change, the standard *k–ε* model offers good numerical robustness and computational efficiency and has been widely adopted in CFD studies of ultrasonic polishing and acoustic streaming. Therefore, within the scope of capturing global flow characteristics, pressure distribution, and cavitation region evolution, the use of the standard *k–ε* model is considered appropriate.

In the standard *k–ε* model, the governing equations for the turbulent kinetic energy and dissipation rate are expressed as follows [41]:

(9) $$\frac{\partial }{\partial t}(\rho k)+\frac{\partial }{\partial x_{i}}(\rho ku_{i})=\frac{\partial }{\partial x_{i}}\left[\left(\mu +\frac{\mu_{i}}{\sigma_{k}}\right)\frac{\partial k}{\partial x_{i}}\right]+G_{k}-\rho \varepsilon$$

(10) $$\frac{\partial }{\partial t}(\rho \varepsilon )+\frac{\partial }{\partial x_{i}}(\rho \varepsilon u_{i})=\frac{\partial }{\partial x_{i}}\left[\left(\mu +\frac{\mu_{i}}{\sigma_{\varepsilon }}\right)\frac{\partial \varepsilon }{\partial x_{i}}\right]+c_{1}G_{k}\frac{\varepsilon }{k}-c_{2}\rho \frac{\varepsilon^{2}}{k}$$

where the turbulent viscosity coefficient is given by $u_{i}=\rho c_{\mu }\frac{k^{2}}{\varepsilon }$, with the model constants taking the following values: $c_{\mu }=0.09$; $c_{1}=1.44$; $c_{2}=1.92$; $\sigma_{k}=1.0$; and $\sigma_{\varepsilon }=1.3$.

### 2.3. Simulation Setup

**Figure 3:**
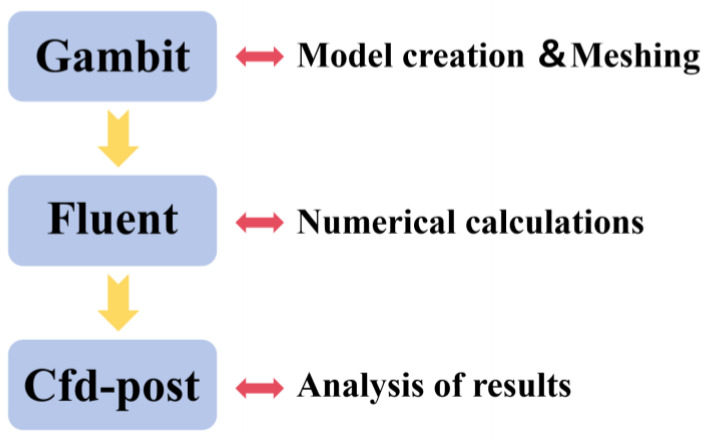
Numerical simulation process.

The numerical simulation process involves simplifying the flow field, constructing the geometric model, generating the computational mesh, performing the numerical calculation, and finally analyzing and outputting the simulation results. The simulation workflow is illustrated in Figure 3.

#### 2.3.1. Geometric Model

**Figure 4:**
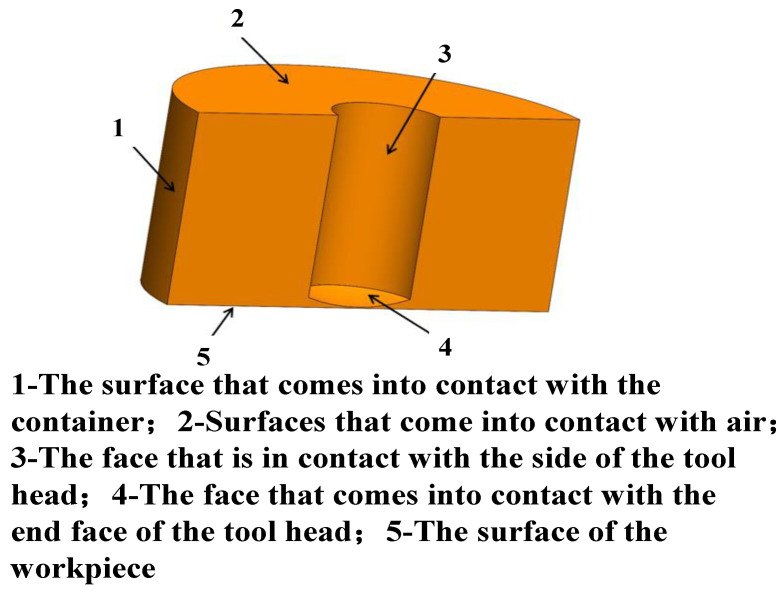
Simplified flow field model of ultrasonic abrasive polishing.

To enable efficient numerical simulations of ultrasonic abrasive polishing, the actual polishing environment must be simplified into a two- or three-dimensional geometric model. This simplification reduces computational complexity, saving both computational resources and time, and facilitates subsequent analysis. Figure 4 shows the simplified flow field model used in this study.

**Figure 5:**
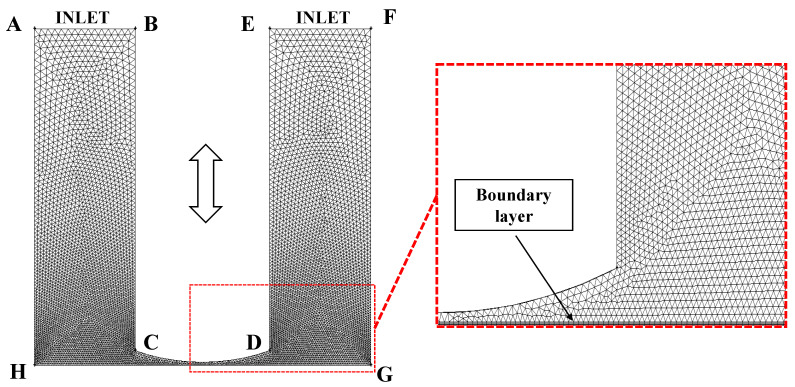
Two-dimensional model and mesh generation.

Given the axisymmetric nature of the flow field, a two-dimensional axisymmetric simplification is justified. Accordingly, a 2D model was constructed and discretized using the pre-processing software GAMBIT 2.4.6. The resulting computational mesh is shown in Figure 5.

Given the curved profile of the polishing tool head and the need to observe its vibration characteristics and the dynamic behavior of the surrounding flow field, mesh refinement was applied in the region adjacent to the tool head. Considering the large flow field gradients and the pronounced variations in cavitation and velocity near the tool head, smaller cell sizes were adopted on the tool head surface and within the nearby gap, with a smooth transition from fine to coarse cells toward the outer-field mesh to improve the accuracy in capturing key flow features and to ensure computational stability. In addition, an unstructured mesh scheme was adopted for grid generation in this study. Compared with structured meshes, the main advantage of unstructured meshes lies in their superior geometric adaptability: they can discretize complex geometric boundaries using arbitrarily shaped elements (e.g., triangles) and readily enable local refinement in critical regions. Moreover, in subsequent simulations involving dynamic mesh techniques, unstructured meshes facilitate the generation of higher-quality remeshed grids during mesh deformation, thereby improving convergence and numerical stability.

In the two-dimensional model depicted in Figure 5, AH and FG represent the internal walls of the container, and HG corresponds to the workpiece surface. The BC, CD, and DE collectively form the polishing tool head with an arc-shaped tip, where segment CD corresponds to the curved surface of the tool head. The AB and EF denote the interfaces between the container interior and the air. The dimensions, physical properties, and boundary condition settings for each edge are summarized in Table 1.

#### 2.3.2. Simulation Parameter Settings

Given the complexity of ultrasonic abrasive polishing, several reasonable simplifications were introduced based on the following assumptions. First, because the abrasive particles are small and uniformly dispersed in the base fluid, the abrasive slurry was treated as a homogeneous liquid phase. Accordingly, the original solid–liquid–vapor three-phase system was simplified to a vapor–liquid two-phase system. Second, gravity was neglected because its effect is insignificant compared with turbulent viscous forces, and viscous dissipation was not considered. Third, the polishing slurry was modeled as a Newtonian fluid and treated as an unsteady incompressible flow, because the base fluids commonly used (e.g., water, kerosene, and methanol) are Newtonian and the viscosity strongly affects polishing performance. Finally, the temperature in the computational domain was assumed to reach equilibrium instantaneously.

### 2.4. Moving Boundary Setting

Since ultrasonic abrasive polishing relies on the ultrasonic vibration of the tool head, a dynamic mesh model must be incorporated into the simulation to characterize this motion. Based on the velocity function of the tool head during ultrasonic abrasive polishing, the vibration frequency and amplitude of the tool head surface (moving wall) are defined via a user-defined function (UDF). The time step size is set to 1/(100f), with a maximum of 100 iterations per time step, meaning each vibration cycle is subdivided into 100 steps, and the results are output at each step to facilitate subsequent visualization and analysis.

The velocity function of the tool head surface during ultrasonic abrasive polishing is typically expressed as a harmonic oscillation formula:

(11) v=A·ω·cos(ω·t)

where ω=2πf, with f representing the vibration frequency; A denotes the amplitude of the tool head; and t represents time. In this simulation, the ultrasonic parameters are set as follows: frequency = 20 kHz; amplitude = 12 μm.

Based on the selected mathematical models and fundamental assumptions adopted for the numerical simulation of ultrasonic abrasive polishing as described above, the key parameters ultimately determined for the simulation are summarized in Table 2.

## 3. Results and Discussion

### 3.1. Analysis of Ultrasonic Flow Field Simulation Results Without the Cavitation Model

**Figure 6:**
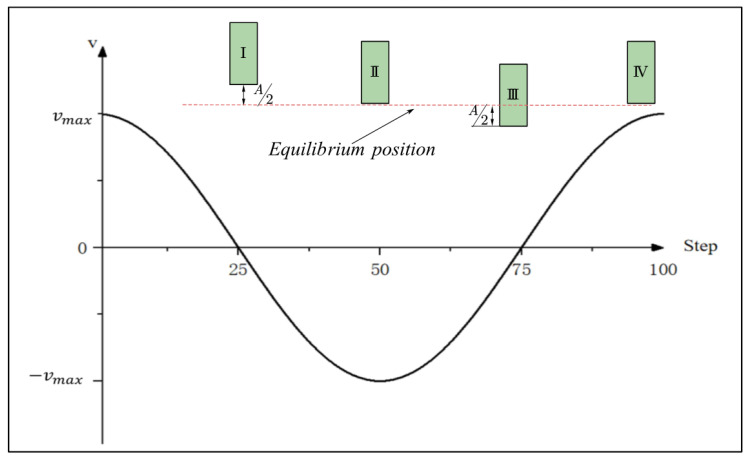
Tool head velocity profile and corresponding tool head positions. I–IV represent the distances of the tool head from the equilibrium position at four key time points.

According to the velocity equation of the tool head surface, the tool head starts moving upward from the equilibrium position at the initial time with the maximum velocity Vmax. By the 25th time step, it reaches position I, as shown in Figure 6, where the distance between the tool head surface and the workpiece surface is maximized. From the 25th to the 75th time steps, the tool head subsequently moves downward from position I through position II to position III, where the gap between the tool head and the workpiece surface is minimized. Finally, (from the 75th to the 100th time step), the tool head returns from position III to the equilibrium position, completing one full cycle of motion. The flow field parameters during this process are analyzed below.

#### 3.1.1. Pressure Analysis

**Figure 7:**
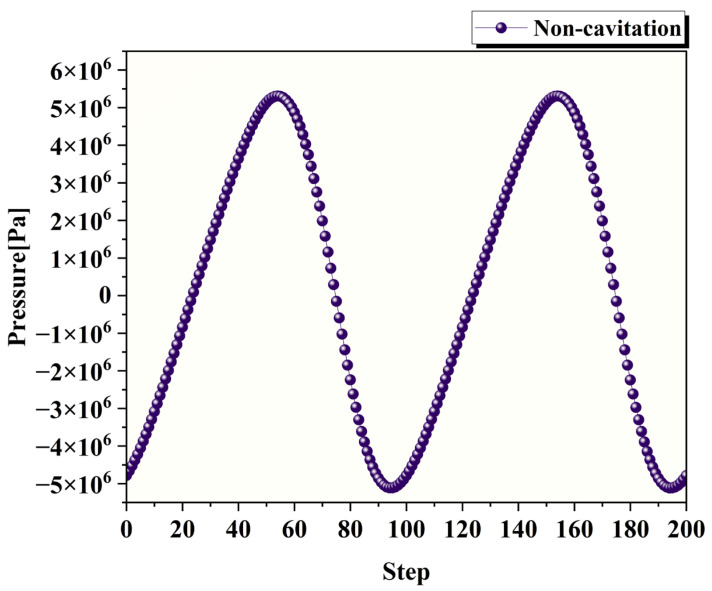
Pressure variation curve at the center point of the processing region.

In the simulation results, four specific time steps (25th, 50th, 75th, and 100th) were selected for analysis, corresponding to four characteristic positions of the tool head movement in Figure 6. Figure 7 shows the pressure variation curve at the center point of the processing region under ultrasonic frequency of 20 kHz, amplitude of 12 μm; gap distance of 72 μm, polishing fluid viscosity of 0.8 Pa·s, density of $1200\;kg/m^{3}$ and without introducing a cavitation model.

As depicted in Figure 7, in the ultrasonic abrasive polishing simulation without a cavitation model, the pressure at the center of the processing region alternates between positive and negative values following the tool head’s motion.

**Figure 8:**
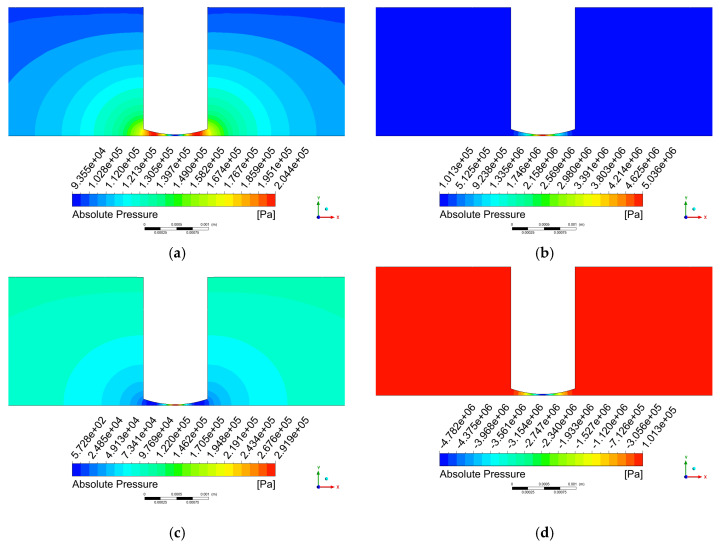
Pressure contour plot on the x-y plane. (**a**) step 25; (**b**) step 50, (**c**) step 75; (**d**) step 100.

Figure 8 displays the pressure contour plot on the x-y plane, where the most intense pressure variations occur directly beneath the tool head. The pressure distribution exhibits relatively regular patterns at the 50th and 100th time steps. At the 50th time step, the central region of the machining zone experiences the highest pressure, with a maximum value of $5.036\times 10^{6}\;Pa$, which decreases radially toward the surrounding flow field.

At the 100th time step, the pressure distribution shows an opposite trend to that at the 50th time step, with the central region exhibiting the minimum pressure value of −$4.782\times 10^{6}\;Pa$, increasing radially toward the periphery. In contrast, the pressure distributions at the 25th and 75th time steps display more distinct characteristics within the flow field.

**Figure 9:**
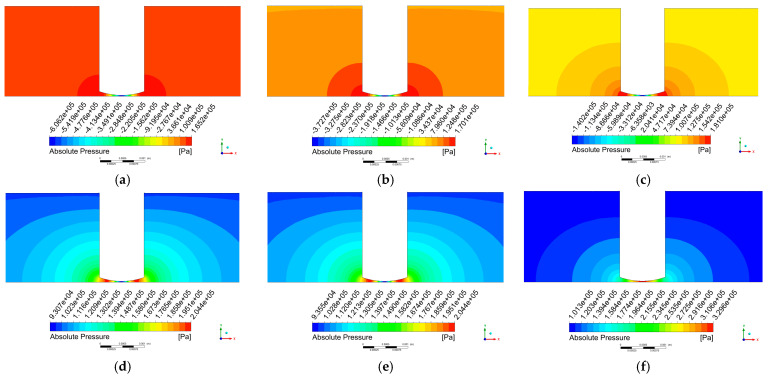
Pressure contour on the x-y plane at steps 21–26. (**a**) step 21; (**b**) step 22; (**c**) step 23; (**d**) step 24; (**e**) step 25; (**f**) step 26.

Analysis of the simulation results from steps 0 to 25 identified an irregular pressure distribution pattern beginning at step 21 and concluding at step 25. Figure 9 presents the pressure contour plots on the x-y plane from steps 21 to 26. The plots show that at step 21, a region of elevated pressure begins to form near the periphery of the tool head. As the polishing process progresses, this high-pressure zone gradually contracts toward the edge of the tool head.

During this process, the continuous expansion of the machining gap leads to the formation of a negative pressure zone at the center of the processing region. Driven by the pressure differential, the surrounding high-pressure fluid flows toward this negative pressure area. As the tool head velocity progressively decreases until it reaches zero at step 25, the machining gap ceases to expand. At this point, the pressure differential driving the fluid flow completely disappears. However, due to inertial effects, the fluid continues to flow toward the processing region, resulting in a subsequent pressure increase. This mechanism is analogous to the positive water hammer effect in fluid dynamics [42].

The pressure distribution pattern at step 75 exhibits characteristics opposite to those at step 25. During steps 71–75, an anomalous pressure distribution appears due to a mechanism similar to that at step 25. When the tool head reaches its lowest position at step 75, the pressure difference driving the outward fluid flow vanishes entirely. Nevertheless, inertial effects cause the fluid to continue moving outward, leading to a pressure reduction near the tool head at step 75. This behavior resembles the negative water hammer effect in fluid dynamics [42].

The pressure distribution exhibits anomalies during steps 21–25 and 71–75 due to the kinematic characteristics of the tool head. During all other time intervals, the pressure distribution consistently follows a gradient pattern that decreases radially from the center of the processing region toward the surrounding flow field.

#### 3.1.2. Speed Analysis

**Figure 10:**
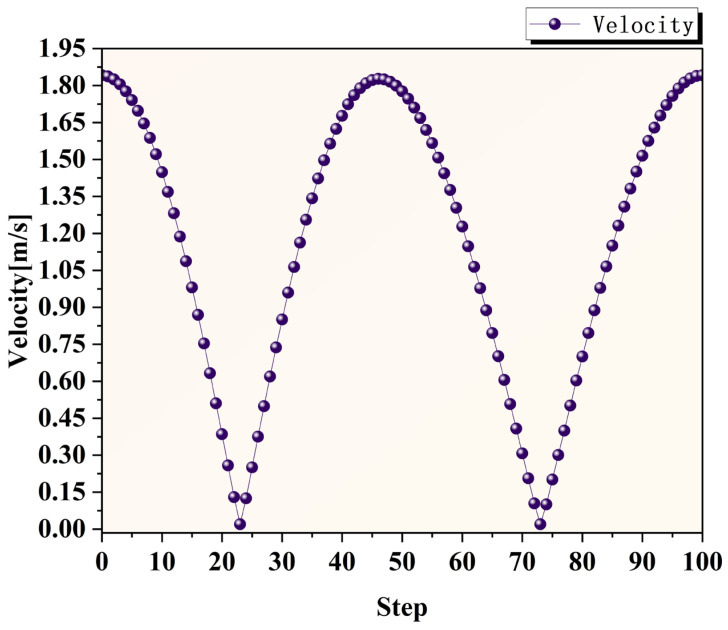
Velocity variation curve near the workpiece surface.

Figure 10 shows the velocity variation curve near the workpiece surface. The temporal evolution of the flow field velocity can be observed from the figure. As illustrated, the overall trend of fluid velocity near the workpiece surface aligns with the variation pattern of the tool head velocity, with the maximum velocity reaching 1.84 m/s and the minimum velocity recorded at 0.01 m/s.

**Figure 11:**
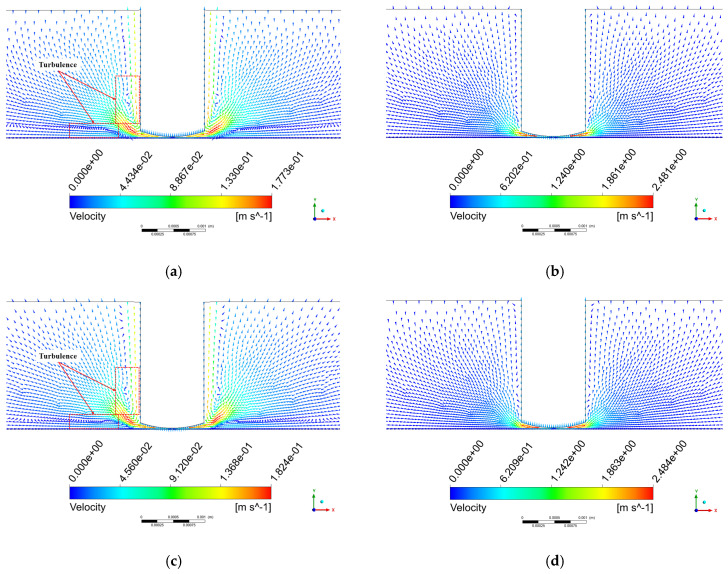
Velocity vector distribution on the x-y plane. (**a**) step 25; (**b**) step 50; (**c**) step 75; (**d**) step 100.

Figure 11 shows the velocity vector distribution on the x-y plane. Figure 11a displays the velocity vectors at step 25, from which it can be observed that the flow velocity at the edge of the tool head is higher than that near the workpiece surface, the flow direction in the central region has changed, and turbulent flow appears along the side of the tool head.

From steps 0 to 25, the tool head is in its upward motion phase, and the polishing gap increases from the initial H = 72 μm to $H_{max}\;=\;72\;\mu m\;+\;A/2$. This process occurs within an extremely short duration, resulting in the instantaneous formation of a negative pressure zone in the flow field. Owing to the significant pressure difference, fluid rapidly converges toward the center of the processing zone, leading to increased flow velocity near the edge of the tool head. As the polishing fluid accumulates in the central region, the flow direction in this area consequently changes. During this stage, the flow field becomes disordered, and turbulent flow emerges along the side of the tool head.

Figure 11b shows the velocity vectors at step 50. The direction of the fluid velocity in this stage is opposite to that at step 25, and the overall velocity distribution is more organized. Between steps 25 and 50, the tool head begins its downward motion after reaching its highest position. As illustrated in Figure 6, during this stage, the tool head moves downward at its maximum velocity, leading to a significant increase in the overall flow field velocity, with the maximum velocity reaching 2.48 m/s.

Figure 11c presents the velocity vectors at step 75. The velocity distribution pattern at this stage is similar to that observed at step 25 but in the opposite direction. During steps 50 to 75, the tool head continues its downward motion until it reaches the lowest position at $H_{min}=72\;\mu m-A/2$. As indicated in Figure 6, the tool head velocity gradually decreases during this phase, resulting in a reduction in the overall flow field velocity. The maximum fluid velocity at this stage is measured at 0.182 m/s.

Figure 11d illustrates the velocity vectors at step 100. During steps 75 to 100, the tool head accelerates upward from its lowest position toward the equilibrium position. As shown in Figure 6, the tool head attains its maximum velocity at step 100, causing a notable increase in fluid velocity. The maximum velocity at this stage is 2.48 m/s, and its distribution pattern resembles that at step 50 but in the opposite direction.

Analysis of the four key time points within one vibration cycle reveals a symmetrical characteristic in the flow field velocity distribution. At steps 25 and 75, the maximum velocities are relatively low (approximately 0.18 m/s), whereas at steps 50 and 100, significantly higher maximum velocities are observed (approximately 2.48 m/s). In all the cases, the maximum velocity values occur at the periphery of the tool head.

### 3.2. Analysis of Ultrasonic Flow Field Simulation Results with the Cavitation Model

#### 3.2.1. Pressure Analysis

In actual processing, cavitation inevitably occurs within the fluid medium. Introducing a cavitation model into the simulation allows for a more accurate representation of actual processing conditions. When the cavitation model is incorporated, significant changes occur in flow field parameters such as pressure, velocity, and vapor fraction. A comparative analysis of simulation results with and without the cavitation model can clearly reveal the dynamic behavior of the flow field and the impact of cavitation on the polishing process.

**Figure 12:**
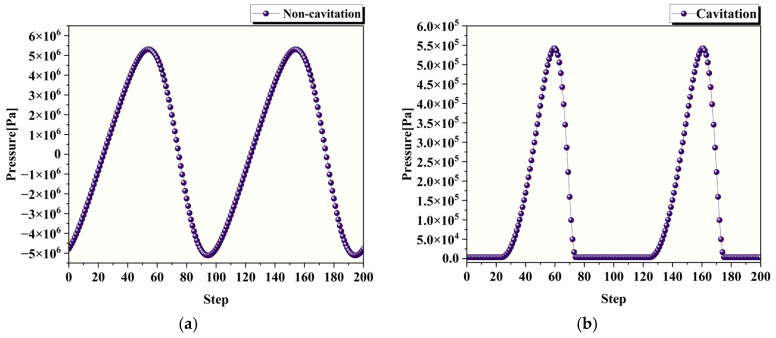
Pressure comparison at the center point of polishing zone. (**a**) Pressure curve without cavitation model; (**b**) Pressure curve with cavitation model.

Figure 12 presents a comparison of the pressure at the center point of the processing zone with and without the cavitation model after the cavitation model is incorporated, while all the other parameters remain consistent. Figure 12a shows the simulation results without the cavitation model, where the flow field exhibits alternating positive and negative pressures, with a maximum pressure of 5,280,071 Pa and a minimum pressure of −5,088,019 Pa. Figure 12b presents the simulation results with the cavitation model. Figure 12b displays the result obtained with the cavitation model. As can be observed, after incorporating the cavitation model, negative pressure no longer appears in the flow field. The minimum pressure remains near the saturation vapor pressure of water (3450 Pa), whereas the maximum pressure is 519,940.125 Pa, which is significantly lower than the maximum pressure observed without the cavitation model.

**Figure 13:**
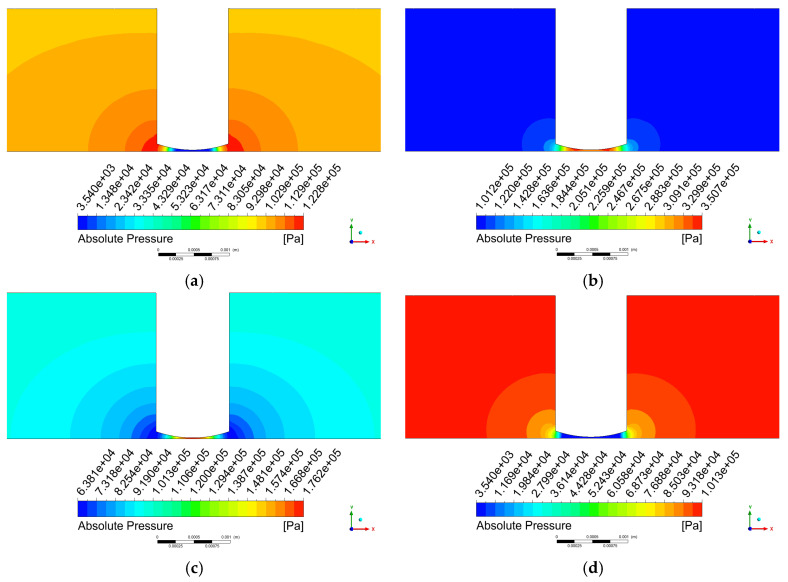
Pressure contour on the x-y plane. (**a**) step 25; (**b**) step 50; (**c**) step 75; (**d**) step 100.

As shown in Figure 13b,d, the pressure distributions at time steps 50 and 100 remain generally consistent regardless of whether the cavitation model is incorporated. The primary distinction observed after introducing the cavitation model lies in the spatial extent of the high- and low-pressure regions. Figure 13a displays the pressure contour at step 25, where a pressure rise occurs near the tool head periphery due to the positive water hammer effect. Figure 13c shows the pressure contour at step 75, showing a pressure reduction around the tool head edge resulting from the negative water hammer effect.

#### 3.2.2. Velocity Analysis

**Figure 14:**
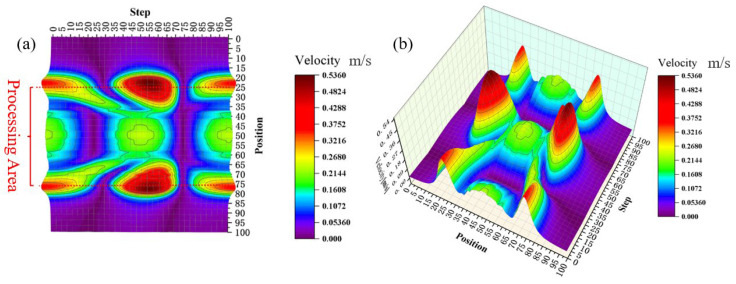
Three-dimensional velocity contour near workpiece surface. (**a**) 2D contour map of velocity magnitude near the workpiece surface; (**b**) 3D distribution of velocity magnitude near the workpiece surface.

Figure 14 presents a three-dimensional velocity contour near the workpiece surface. Compared with the results without the cavitation model, the inclusion of cavitation reduces the maximum velocity at the tool head edge from 1.84 m/s to 0.536 m/s, while the minimum velocity remains consistent. Furthermore, the velocity distribution in the flow field becomes irregular, although the maximum velocity continues to occur at the tool head edge.

**Figure 15:**
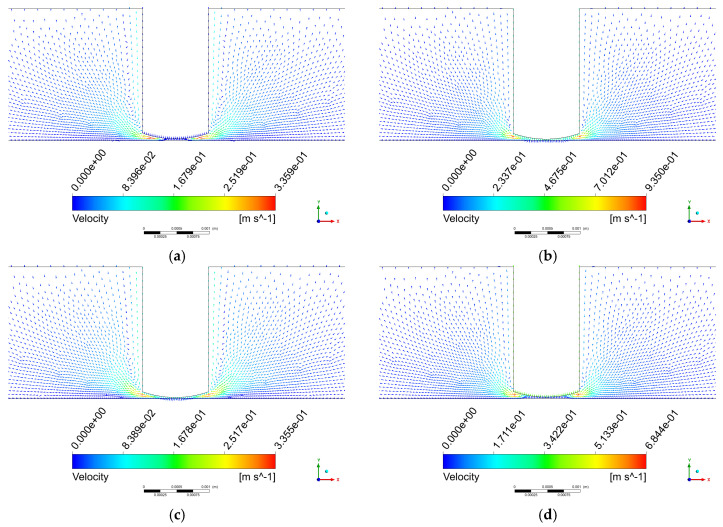
Velocity vector distribution on the x-y plane. (**a**) step 25; (**b**) step 50; (**c**) step 75; (**d**) step 100.

Figure 15 shows the velocity vector distribution on the x-y plane. In contrast to the flow field velocity distribution without the cavitation model, the velocity direction in the center of the machining zone shifts to a vertical orientation after introducing the cavitation model, while the maximum velocity still appears at the periphery of the tool head.

#### 3.2.3. Vapor Fraction Analysis

**Figure 16:**
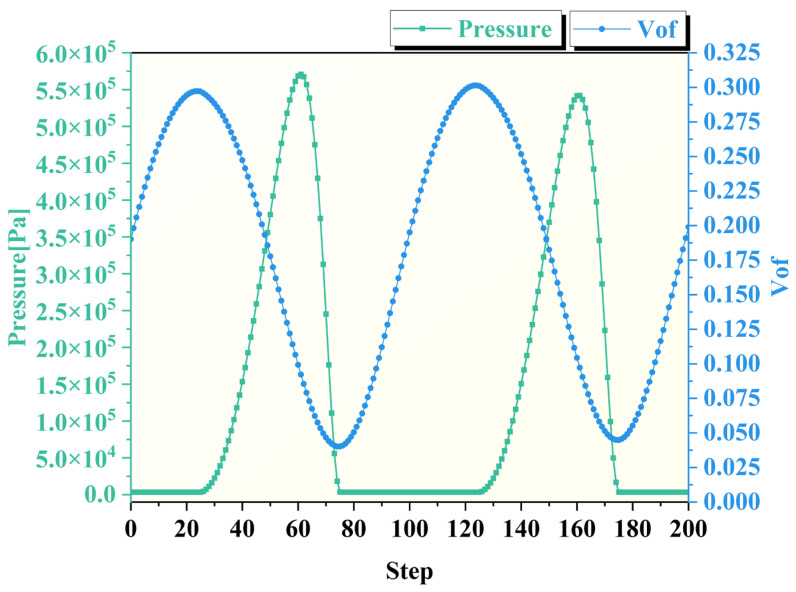
Variation curves of absolute pressure and vapor fraction on the workpiece surface.

As previously mentioned, the full cavitation model serves as a core tool in the simulation parameter settings for modeling cavity formation when the local liquid pressure falls below the saturation vapor pressure (3450 Pa). The pressure analysis indicates that the alternating high and low pressures at the center of the machining region directly promote cavitation, which occurs predominantly in this area. Figure 16 shows the extracted pressure and vapor fraction curves at the center point of the machining zone. An inverse relationship is observed between absolute pressure and vapor fraction, both exhibiting periodic variations: as pressure decreases, vapor bubbles grow; as pressure increases, the bubbles compress and collapse.

**Figure 17:**
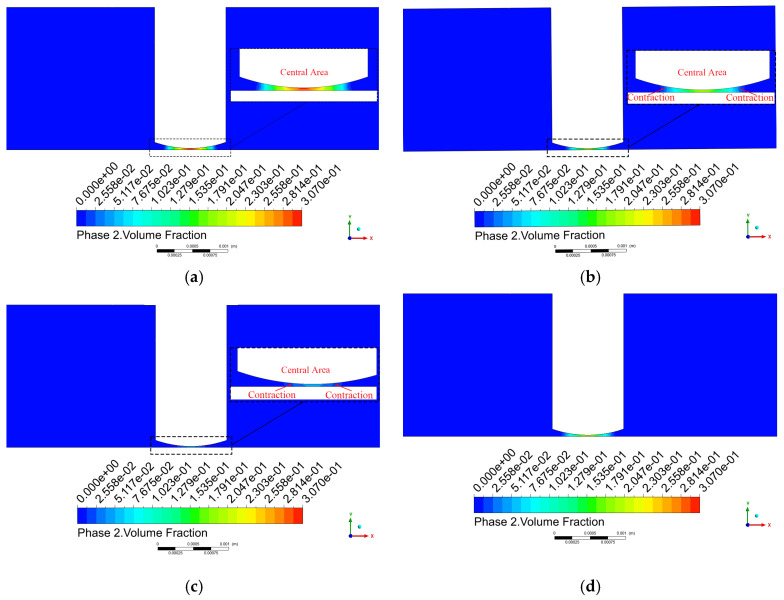
Vapor volume fraction distribution on the x-y plane. (**a**) step 25; (**b**) step 50; (**c**) step 75; (**d**) step 100.

Figure 17 shows the vapor volume fraction distribution on the x-y plane. From steps 25 to 75, cavitation bubbles gradually dissipate, with both the extent and volume fraction of the bubbles significantly diminishing. Notably, during this period, the cavitation bubbles continuously shrink toward the side of the tool head, while those on the workpiece surface diminish earlier. This indicates that cavitation does not occur uniformly throughout the flow field but is primarily concentrated near the workpiece surface and adjacent regions.

**Figure 18:**
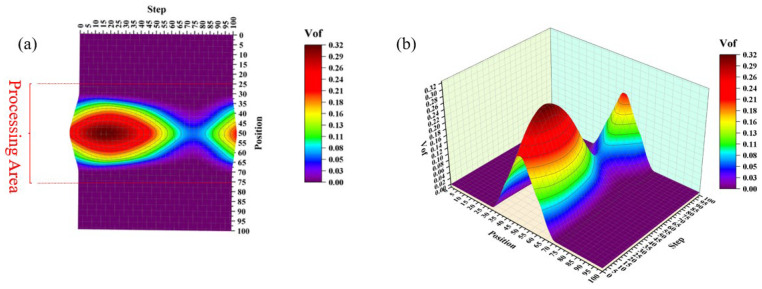
Three-dimensional contour of vapor volume fraction variation on workpiece surface. (**a**) 2D contour map of vapor volume fraction (VoF) near the workpiece surface; (**b**) 3D distribution of vapor volume fraction (VoF) near the workpiece surface.

As shown in Figure 18, during the downward motion of the tool head, the number of cavitation bubbles continuously decreases, and the area where cavitation occurs gradually diminishes. By step 75, the number of cavitation bubbles essentially stabilizes but does not completely disappear. This phenomenon arises because the duration of the pressure increase is insufficient, leading to incomplete cavitation phenomena in the flow field.

A comprehensive analysis of the pressure, velocity, and vapor fraction distributions in the flow field after introducing the cavitation model leads to the following conclusions. Cavitation modifies the pressure distribution, and the resulting pressure variation is the primary cause of the observed changes in both the velocity field and the vapor fraction. The flow velocity near the periphery of the tool head is higher than that in the central region, and the tangential velocity component exceeds the normal component. However, the pressure reduction induced by cavitation causes an overall decrease in flow velocity and may also introduce cavitation-erosion pits, which are detrimental to polishing quality. Therefore, subsequent process optimization should be formulated in a goal-oriented manner: for finishing operations targeting low-defect, high-quality surfaces, excessive cavitation—predominantly occurring in the central region—should be suppressed, whereas for applications prioritizing high removal rate or surface strengthening, a controlled-cavitation strategy can be adopted, followed by a low-cavitation finishing step to mitigate pit defects. Cavitation intensity can be regulated by adjusting process parameters, such as the ultrasonic polishing gap and the base-fluid viscosity [22,23,26].

## 4. Ultrasonic Abrasive Polishing Experiment

### 4.1. Workpiece Material and Polishing Suspension

The experimental material consisted of 1Cr17 stainless steel specimens with a diameter of 20 mm and thickness of 5 mm. 1Cr17 is a typical ferritic stainless steel, whose chemical composition and fundamental mechanical properties are summarized in Table 3. Prior to testing, all samples were uniformly ground with sandpaper to achieve a consistent surface roughness of Ra = 0.4 μm, facilitating subsequent observation and evaluation of processing outcomes. The polishing suspension employed silicon carbide (SiC) abrasives with a grit size of 600 mesh (approximately 23 μm). The polishing fluid was formulated with a viscosity of 0.8 mPa·s and a density of 1200 kg/m^3^, maintaining consistency with the simulation parameters.

### 4.2. Equipment Introduction

**Figure 19:**
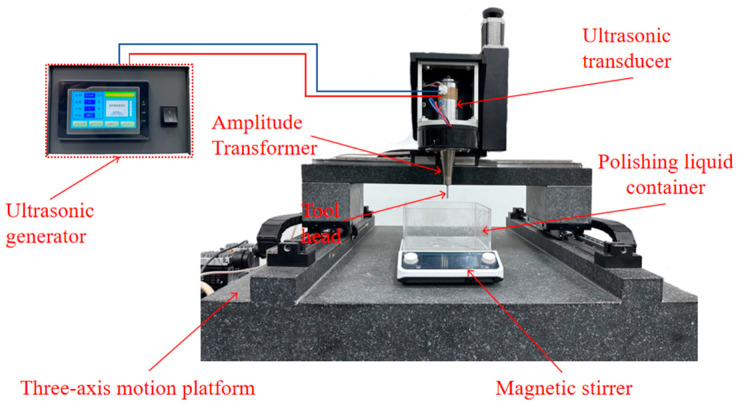
Experimental setup.

The experimental setup was implemented on a custom high-precision three-axis motion platform, powered by an ultrasonic generator operating at a frequency of 20 kHz with an adjustable amplitude range of 0–40 μm, as shown in Figure 19. The processed surface was examined using a three-dimensional laser confocal microscope (Keyence VK-1000, Osaka, Japan), while changes in residual stress on the workpiece surface were measured with an X-ray stress analyzer (HDS-1).

### 4.3. Experimental Design

The experimental parameters were consistent with the simulation conditions, with specific details provided in Table 4. To minimize errors, each experimental group was processed four times with durations of 120 s, 150 s, and 180 s, respectively. By comparing the surface machining marks on the workpiece under different machining times and analyzing the residual stresses at various locations within the machining zone, the material removal mechanism was investigated.

### 4.4. Analysis of the Experimental Results

In sub-micrometer ultra-precision machining processes, the geometric morphology and mechanical characteristics of machining marks not only influence the material removal mechanism but also exert a decisive impact on the precision, integrity, service performance, and lifespan of the final machined surface. Therefore, systematic analysis of the morphological characteristics, distribution patterns, mechanical properties, and underlying causes of machining marks constitutes a critical prerequisite for optimizing process parameters and improving machining quality.

#### 4.4.1. Surface Morphology Examination

**Figure 20:**
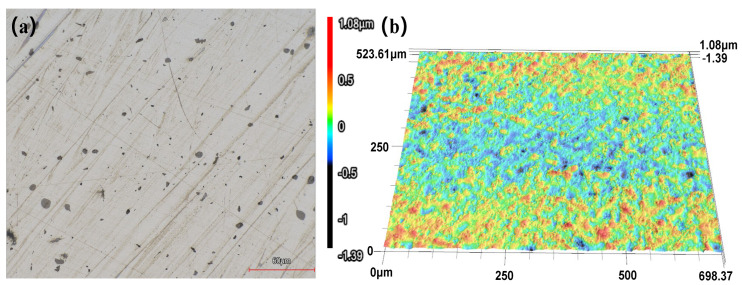
Workpiece surface before machining. (**a**) Two-Dimensional Image (**b**) Surface Morphology.

Figure 20 presents the surface morphology and corresponding 3D images of the workpiece after manual polishing. As shown in Figure 20a, despite the manual polishing process, the workpiece surface still exhibits numerous scratches and fine indentations.

**Figure 21:**
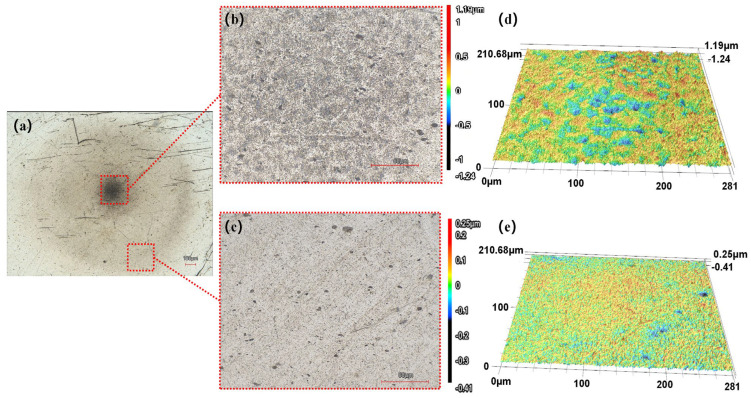
Experimental results after 120 s of processing. (**a**) Overview 2D image (**b**) 2D image of the central region (**c**) 2D image of the edge region (**d**) Surface morphology of the central region (**e**) Surface morphology of the edge region.

Figure 21 shows the machining marks remaining on the workpiece surface after 120 s of processing. Distinctly different processing effects can be observed at various locations within the machining zone. Processing marks are more pronounced at the center and edges of the machining area, while a region with weaker processing outcomes is noted between the center and the edges. Figure 21b,c present magnified views of the center and edge of the processed area after 120 s of machining, respectively. Figure 21d,e display their corresponding three-dimensional images. As shown in Figure 21b, the center of the machining zone has darkened noticeably, with irregularly distributed pits appearing, and the marks left by prior manual polishing have significantly diminished. This phenomenon is attributed primarily to the concentrated occurrence of cavitation in the central region of the machining zone. Under the impact of micro-jets generated by cavitation and the abrasive particles driven by these jets, plastic deformation occurs on the workpiece surface, resulting in the formation of pockmarks and honeycomb-like pores.

Figure 21c reveals a noticeable reduction in scratches and indentations in the edge region, owing to the scouring and polishing action of the high-speed flowing polishing fluid in this area. The processing marks generated by the polishing effect of the rapidly flowing fluid are shallower than those induced by cavitation, leading to a lower material removal rate. Although scratches from previous polishing remain visible, they have improved considerably compared to the unprocessed state, with the edges of these marks becoming blurred.

**Figure 22:**
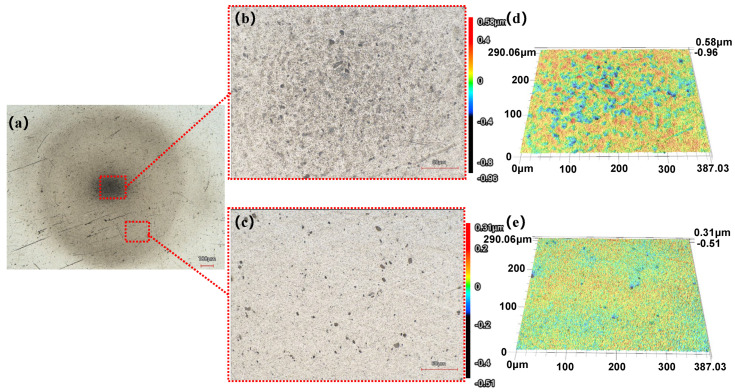
Experimental results after 150 s of processing. (**a**) Overview 2D image (**b**) 2D image of the central region (**c**) 2D image of the edge region (**d**) Surface morphology of the central region (**e**) Surface morphology of the edge region.

**Figure 23:**
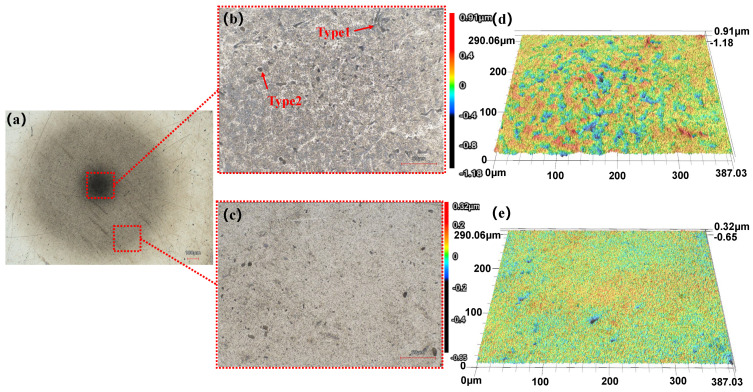
Experimental results after 180 s of processing. (**a**) Overview 2D image (**b**) 2D image of the central region (**c**) 2D image of the edge region (**d**) Surface morphology of the central region (**e**) Surface morphology of the edge region.

Figure 21b, Figure 22b and Figure 23b reveal that the damaged area on the workpiece surface gradually expands with increasing processing time. This phenomenon is attributed primarily to continuous erosion caused by cavitation effects at the center of the machined area. Furthermore, in the experimental results for a machining time of 180 s (Figure 23b), numerous distinct pits can be observed in the central region, which can be categorized into two types on the basis of their morphology and formation mechanisms. One is irregular pits formed by high-frequency impact of microjets and shockwaves generated during the collapse of cavitation bubbles (labeled Type 2 in Figure 23b). The other is irregular wedge-shaped pits resulting from the impact of abrasive particles accelerated by cavitation microjets (labeled Type 1 in Figure 23b).

Figure 21c, Figure 22c and Figure 23c display the surface morphology at the edge of the processed area under three machining durations. At the shorter machining time of 120 s, scratches remain clearly visible at the edge. As the time increases to 150 s, most scratches are removed, leaving only a few deeper ones. When the processing time is further extended to 180 s, scratches in the edge region are completely eliminated. Thus, the results demonstrate non-uniform distribution of processing effects, with significantly more pronounced material removal observed in the central region compared to the relatively superficial outcomes at the edge. The experiments indicate that the cavitation effect in the machining zone has a greater influence on material removal than the polishing and grinding action of the polishing fluid. However, cavitation introduces new surface pits and defects, necessitating further research to develop effective suppression strategies.

#### 4.4.2. Residual Stress Measurement

**Figure 24:**
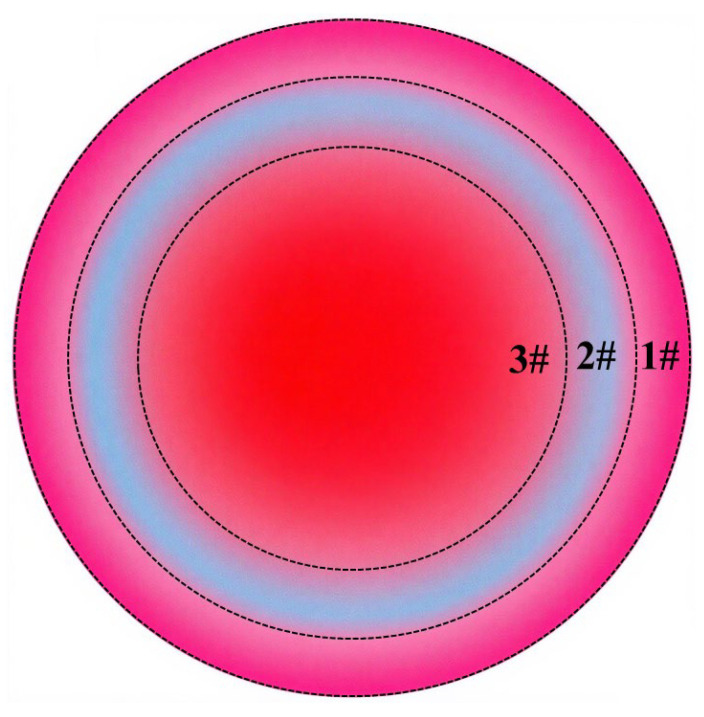
Schematic diagram of residual stress measurement areas.

Residual stress measurements were conducted at four points across three distinct zones on the machined workpiece surface, including the center zone, the transition zone between the center and the edge, and the machined edge zone. The average value of these four measurements was calculated to represent the residual stress for each respective zone. As shown in Figure 24, labels #3, #2 and #1 indicate the center zone, the transition zone and the machined edge zone, respectively.

**Figure 25:**
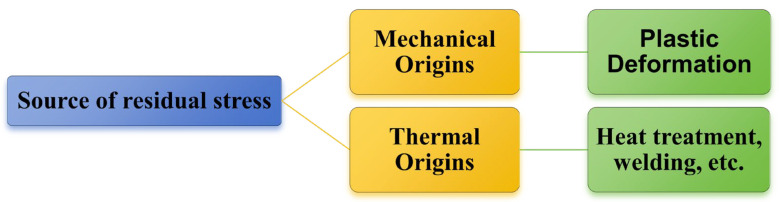
Sources of residual stress.

Residual stresses on workpiece surfaces originate from two primary sources: thermal and mechanical origins, as illustrated in Figure 25. Thermally induced residual stresses arise mainly from incompatible thermal deformations between different regions or components caused by thermodynamic changes such as temperature gradients, thermal cycles, or phase transformations during manufacturing or processing. Mechanically induced residual stresses, on the other hand, result from inhomogeneous plastic deformation of the material due to external forces during mechanical processing. For instance, mechanical actions like cutting, grinding, or extrusion can cause lattice distortion, dislocation multiplication, and rearrangement, leading to a state of stress imbalance in the surface layer of the material [43].

Residual compressive stresses induced on workpiece surfaces by ultrasonic abrasive polishing arise from the synergistic effects of cavitation-induced shock waves and the high-frequency impact of abrasive particles excited by these shock waves. This mechanism produces substantial plastic deformation in the surface layer of the material. The resulting volumetric changes in the surface layer are constrained by the underlying core material, ultimately leading to the formation of residual compressive stresses on the workpiece surface. This residual compressive stress effectively counteracts the tensile stress induced by external cyclic loading, suppresses plastic strain in the metallic material, and hinders the initiation and propagation of cracks, thereby contributing positively to the enhancement of the material’s fatigue life [44,45].

**Figure 26:**
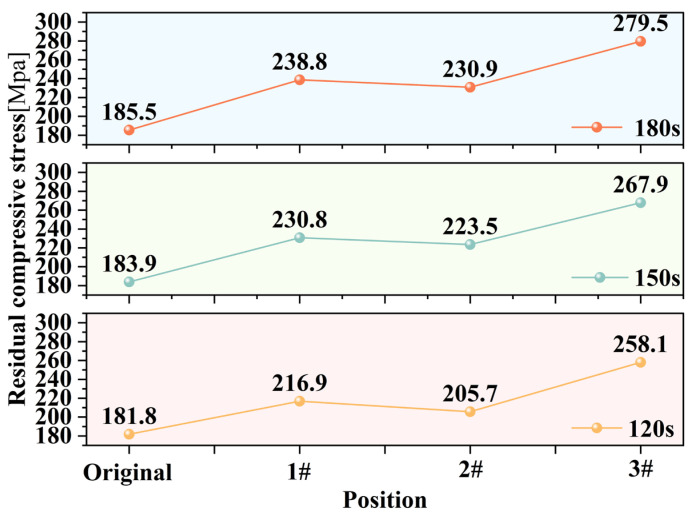
Residual compressive stress at different processing times and locations.

As illustrated in Figure 26, the evolution of residual stress across different surface regions was quantified following fixed-point ultrasonic abrasive polishing for varying durations. All specimens exhibited initial compressive residual stresses due to prior manual polishing pretreatment, with consistent baseline values averaging approximately 180 Mpa. Post-processing measurements revealed increased compressive stress levels across all zones, showing distinct regional variations. Notably, the magnitude of compressive stress demonstrated a positive correlation with processing time, indicating time-dependent stress accumulation.

Based on the aforementioned analysis, the polishing fluid exhibits distinctly different dynamic behaviors in various regions of the machining area during fixed-point ultrasonic abrasive polishing. This variation leads to differences in material removal mechanisms at different locations within the processed zone.

Intense cavitation occurring at the center of the processing zone generates microjets and shock waves that impose high-frequency mechanical impacts on the workpiece surface. Simultaneously, this process causes abrasive particles to impact the surface at high velocities, inducing severe plastic deformation in the workpiece. Under such high-intensity impact, the surface residual compressive stress progressively increased with extended processing time, rising from an initial value of approximately 180 Mpa to 258.1 Mpa, 267.9 Mpa, and finally reaching 279.5 Mpa. The stress enhancement magnitude continues to increase with processing duration: 29.6% after 120 s, 31.4% after 150 s, and 33.6% after 180 s of processing.

At the edge of the processing zone, the high-speed flow of polishing fluid drives abrasive particles to impact and scratch the workpiece surface. However, the energy generated by abrasive impact and scratching is substantially lower than that produced by cavitation bubble collapse. Consequently, although there is a noticeable increase in residual stress at the edge region, its magnitude is significantly smaller than in the central area, increasing by 16.2% after 120 s, 20.3% after 150 s, and reaching 22.3% after 180 s of processing.

In the transition zone, the increase in residual compressive stress results from the combined effects of cavitation and mechanical polishing by the polishing fluid. However, since neither material removal mechanism fully dominates this region, the residual stress increase remains slightly lower than that in both the central and edge zones. The measured stress increases at the three processing durations were 11.6%, 17.7%, and 19.7%, respectively.

The distribution of residual stress on the workpiece surface further confirms the existence of two distinct material removal mechanisms during the polishing process, i.e., cavitation erosion-dominated removal in the central region and mechanical abrasion by abrasive particles carried in the high-speed flow of polishing fluid at the edge zone. The former achieves material removal through repeated impacts of microjets and shock waves generated by cavitation bubble collapse, causing fatigue-induced material spalling. The latter accomplishes material removal primarily through the cutting action of abrasive particles.

### 4.5. Material Removal Mechanism

Simulation and experimental results indicate the coexistence of two distinct fluid behaviors within the machining zone: mechanical abrasion induced by high-speed shear flow around the tool periphery and surface erosion caused by cavitation phenomena concentrated in the central region. Over a complete ultrasonic vibration cycle, the tool motion can be divided into the upward movement stage and the downward movement stage.

**Figure 27:**
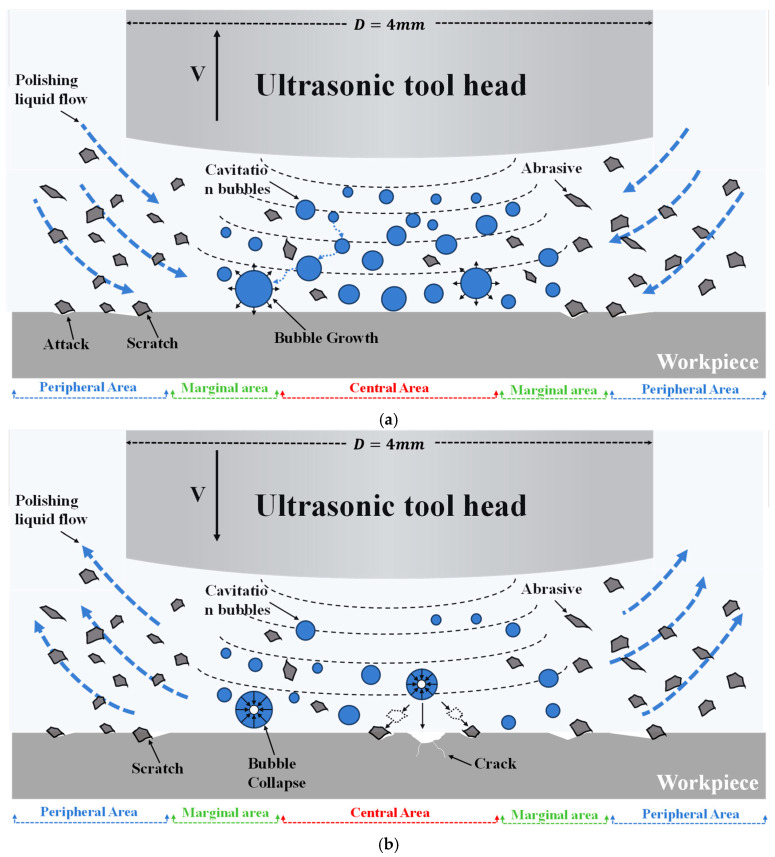
Material removal mechanism during a vibration cycle. (**a**) Upward stroke of the tool head; (**b**) Downward stroke of the tool head.

As shown in Figure 27a, during the upward stroke of the tool head, the polishing fluid flows into the machining zone at a specific angle driven by pressure differences. Within this process, abrasive particles in the fluid impact and scratch the workpiece surface at corresponding angles, achieving mechanical polishing. Simultaneously, a large number of microscopic vapor bubbles are generated in the central region of the machining area due to the low-pressure environment and continue to expand under sustained low-pressure conditions. At this stage, mechanical polishing plays a dominant role in material removal.

Figure 27b shows the downward stroke of the tool head. During this phase, the polishing fluid is squeezed outward by the tool head, while the abrasive particles in the fluid simultaneously polish the workpiece surface. As pressure increases within the processing zone, numerous microscopic bubbles accumulate in the central region during the previous phase collapse. The instantaneous collapse of these cavitation bubbles generates tremendous impact forces. Under the influence of these forces, the abrasive particles surrounding the bubbles are accelerated toward the workpiece surface, causing scratching and erosion.

Furthermore, cavitation erosion constitutes a form of low-cycle fatigue damage. The continuous high-frequency impacts produced by the collapse of a large number of cavitation bubbles within an extremely short duration promote the initiation and propagation of cracks on the workpiece surface, ultimately leading to fatigue fracture and material spallation. During this stage, both mechanical polishing and cavitation erosion contribute synergistically to the material removal process.

## 5. Conclusions

This study combines CFD simulations with experiments to elucidate flow field characteristics and material removal in ultrasonic abrasive polishing. We simulated polishing-gap flow and cavitation and conducted ultrasonic fixed-point polishing with surface morphology and residual stress characterization for validation. The main conclusions are as follows:

1. Significant interactions exist among the flow field parameters in ultrasonic polishing: the pressure distribution drives the initiation and evolution of cavitation, while the resulting phase change and bubble dynamics in turn affect pressure stability and the spatial distribution of bubbles. As a result, cavitation is mainly concentrated at the center of the processing zone, which is identified as the key cause of the spatial non-uniformity in polishing performance.
2. Material removal shows clear spatial zoning:

I. Central zone: Cavitation erosion dominates. Bubble collapse generates microjets and shock waves, causing fatigue spalling and plastic indentation and forming numerous pits. The residual compressive stress increases the most (33.6%), which is beneficial for improving fatigue life and wear resistance.

II. Edge zone: Abrasive mechanical action driven by shear flow dominates. High-speed shear flow carries abrasives to scratch and grind the surface, removing defects and improving surface finish. The residual stress increase is 22.3%, mainly due to near-surface plastic deformation.

III. Transition zone: The two mechanisms compete and superimpose. The removal and surface modification are intermediate, leading to the smallest residual stress increase (19.7%).

Based on these findings, a comprehensive removal model is proposed: tool head vibration excites a transient flow field; cavitation and shear actions are induced; and cavitation impacts and shear flow accelerate abrasive particles, promoting fatigue spalling and material removal.

## Figures and Tables

**Table 1:** Boundary Settings of the Computational Model.

Boundary	Length (mm)	Property	Type
AB	3	Inlet	Pressure inlet
BC	14.45	Deforming	Wall
CD	4.08	Moving wall	Wall
DE	14.45	Deforming	Wall
EF	3	Inlet	Pressure inlet
FG	15	Wall	Wall
GH	10	Workpiece	Wall
HA	15	Wall	Wall

**Table 2:** Model solution parameter.

Model	Dimension	Turbulent	Multiphase	Fluid	Time Step	Monitor
Unsteady	Two	Stand *k–*ε	Mixture	Incompressible	0.5 μs	Pressure-Based

**Table 3:** Chemical composition and mechanical properties of 1Cr17 stainless steel.

Element (wt.%)	C 0.12	Si 1.00	Mn 1.00	P 0.035	S 0.030	Cr 17.54	Ni 0.60	Fe Bal.
Mechanical Properties	Tensile strength /MPa 450	Yield strength /MPa 205	Elongation /% 22	Section shrinkage A /% 50	Impact /J 50	Hardness /HBW 183		

**Table 4:** Parameters for point polishing experiments.

Project	Value
Frequency	20 kHz
Amplitude (peak to peak)	12 μm
Processing gap	72 μm
Viscosity of the polishing liquid	0.8 Pa·s
Density of polishing liquid	$1200\;\mathrm{kg}/m^{3}$
Abrasive content	15 wt%
Abrasive particle size	600 mesh
Polishing Time	120 s,150 s,180 s,
Tool head diameter	4 mm

## Data Availability

The original contributions presented in this study are included in the article. Further inquiries can be directed to the corresponding author.
